# Pedal cadence does not affect muscle damage to eccentric cycling performed at similar mechanical work

**DOI:** 10.3389/fphys.2023.1140359

**Published:** 2023-03-10

**Authors:** Hisashi Ueda, Riki Saegusa, Yosuke Tsuchiya, Eisuke Ochi

**Affiliations:** ^1^ Faculty of Healthcare and Medical Sports, Teikyo Heisei University, Chiba, Japan; ^2^ Faculty of Bioscience and Applied Chemistry, Hosei University, Tokyo, Japan; ^3^ Center for Liberal Arts, Laboratory of Health and Sports Sciences, Meiji Gakuin University, Yokohama, Japan; ^4^ Graduate School of Sports and Health Studies, Hosei University, Tokyo, Japan

**Keywords:** pedaling velocity, total work, work volume, maximal voluntary concentric contraction torque, joint flexibility, muscular dysfunction, muscle damage

## Abstract

**Purpose:** This study aimed to investigate muscle damage when performing equal mechanical work of fast and slow pedaling speed by eccentric muscle actions (ECCs) cycling.

**Methods:** Nineteen young men [mean ± standard deviation (SD) age: 21.0 ± 2.2 years; height: 172.7 ± 5.9 cm; and body mass: 70.2 ± 10.5 kg] performed maximal effort of ECCs cycling exercise with fast speed (Fast) and slow speed trials (Slow). First, subjects performed the Fast for 5 min by one leg. Second, Slow performed until the total mechanical work was equal to that generated during Fast other one leg. Changes in maximal voluntary isometric contraction (MVC) torque of knee extension, isokinetic pedaling peak torque (IPT), range of motion (ROM), muscle soreness, thigh circumference, muscle echo intensity, and muscle stiffness were assessed before exercise, and immediately after exercise, and 1 and 4 days after exercise.

**Results:** Exercise time was observed in the Slow (1422.0 ± 330.0 s) longer than Fast (300.0 ± 0.0 s). However, a significant difference was not observed in total work (Fast:214.8 ± 42.4 J/kg, Slow: 214.3 ± 42.2 J/kg). A significant interaction effect was not observed in peak values of MVC torque (Fast:1.7 ± 0.4 Nm/kg, Slow: 1.8 ± 0.5 Nm/kg), IPT, muscle soreness (Fast:4.3 ± 1.6 cm, Slow: 4.7 ± 2.9 cm). In addition, ROM, circumference, muscle thickness, muscle echo intensity, and muscle stiffness also showed no significant interaction.

**Conclusion:** The magnitude of muscle damage is similar for ECCs cycling with equal work regardless of velocity.

## Introduction

Eccentric muscle actions (ECCs), in which the muscles become tensioned while being stretched, could cause by resulting in micro damage to the sarcomere or an inflammatory response, decreased muscle strength, limited flexibility, delayed onset muscle soreness (DOMS), muscle swelling, increased muscle stiffness, creatine kinase (CK), myoglobin (Mb), and interleukin (IL)-6 in blood. (Clarkson et al., 1992; Morgan and Allen, 1999; Chen et al., 2007; Ochi et al., 2016; Tsuchiya et al., 2019). The degree of muscle damage is caused by ECCs depends on the exercise duration, length, intensity (Nosaka and Sakamoto, 2001; Nosaka and Newton, 2002), repetitions (Hesselink et al., 1996; Chen and Nosaka, 2006) and velocity (Chapman et al., 2006; Chapman et al., 2008a; Ueda et al., 2020).

Several studies have examined the effect of velocities of ECCs on muscle damage (Paddon-Jones et al., 2005; Chapman et al., 2006; Chapman et al., 2008a). In ECCs of elbow flexors, the fast velocity (210°/sec) resulted in greater torque deficit, increased DOMS, upper arm swelling and increased blood CK than the slow velocity (30°/sec) (Chapman et al., 2006). However, the number of contractions in this study differed significantly because the contraction times were matched between the fast and slow conditions (210 contractions in the fast condition and 30 contractions in the slow condition). Therefore, Chapman et al. (2008a) compared muscle damage after ECCs in elbow flexion at different contraction velocities under the condition of similar number of contractions. The results showed that the degree of muscle damage was greater in the fast (210°/sec) than in the slow (30°/sec) even when the number of contractions were combined (210 contractions). Similarly, Barreto et al. (2019) reported that slow-velocity ECCs in elbow flexion presented faster recovery of muscle strength and less muscle soreness compared with high-velocity. Therefore, it showed that the degree of muscle damage in ECCs due to elbow flexion is greater in the fast condition than in the slow, even when the contraction time and number of contractions were standardized. However, the total work was not exactly the same in the previous studies.

Long-term ECCs cycling training improved muscle strength and increased muscle hypertrophy despite being less demanding on the respiratory circulatory system than concentric cycling training (LaStayo et al., 2000). Therefore, it has been reported to be an effective exercise for the elderly, obese and patients with chronic obstructive pulmonary disease (COPD) (Lastayo et al., 1999; LaStayo et al., 2000; Gonzalez-Bartholin et al., 2019; Julian et al., 2019; Nickel et al., 2020). As acute response to ECCs cycling, we investigated the effects of different velocities on muscle damage in one bout of ECCs cycling (Ueda et al., 2020). Our results showed that muscle damage and delayed onset muscle soreness were significantly greater in the fast (210°/sec) than in the slow (30°/sec) condition (Ueda et al., 2020). Therefore, similar to the above findings in elbow flexors, we concluded that the fast ECCs exercise causes greater muscle damage than the slow. However, a limitation of this study is that the workload (365.7 ± 60.6 W) of the fast was significantly greater than that of the slow (68.3 ± 26.6 W) because the exercise duration was unified to 5 min. Thus, it remains unclear which factor, out of pedaling speed or workload, affected the differences in the degree of muscle damage. Contrarily, previous studies on animals have reported that the torque exerted during ECCs exercise and workload is a factor that determines the magnitude of muscle damage (Lieber and Friden, 1993; Warren et al., 1993; Talbot and Morgan, 1998). However, studies examining the effects of differences in velocity on muscle damage in humans under similar workload have been lacking.

Therefore, the purpose of this study was to compare the muscle damage caused by fast-speed (210°/sec) and slow-speed (30°/sec) ECCs cycling under equal the mechanical work (J) conditions. We hypothesized that ECCs cycling motion with different pedaling speeds had the same degree of muscle damage under uniform workload condition.

## Materials and methods

### Subjects

Nineteen young men were recruited (mean ± standard deviation (SD) age: 21.0 ± 2.2 years; height: 172.7 ± 5.9 cm; and body mass: 70.2 ± 10.5 kg). None of the subjects had participated in any regular resistance training for at least 1 year prior to this study. The participants were requested to avoid participation in other clinical trials and interventions, such as hot and cold baths, massage, stretching, strenuous exercise, excessive food, or alcohol consumption, and taking any supplement or medication at least 3 months before and during this trial. All subjects were provided with detailed explanations of the study protocol prior to participation and signed an informed consent form in accordance with the Declaration of Helsinki before being enrolled in this study. Written informed consent was obtained from the individual for the publication of any potentially identifiable images or data included in this article. This study was approved by the Ethics Committee for Human Experiments at Teikyo Heisei University (ID: R01-058-2).

### Experimental protocols

The subjects randomly performed maximal-effort ECCs cycling exercise are unilateral by each leg. ECCs cycling exercises were randomly performed on the same day by the non-dominant leg or dominant leg. First, all subjects performed the fast velocity session (Fast) for 5 min. Second, the slow velocity session (Slow) was reached when the total mechanical work done was equal to that generated during Fast (defined as ‘‘mechanical work”), which was also automatically calculated by ECCs cycling. Previous studies have reported that the initial bout of maximal eccentric muscle actions is responsible for conferring protective effects to the contralateral side (Howatson and van Someren, 2007; Xin et al., 2014; Chen et al., 2016; Tsuchiya et al., 2018). We had set the interval between the slow and fast velocities to 15 min, as this effect occurs when the second bout is performed from 1 day to 4 weeks (Chen et al., 2016). The legs were randomly assigned using a table of random numbers to minimize the intergroup differences in terms of age, body weight, and body mass index (BMI). The dependent variables included maximal voluntary isometric contraction (MVC) torque of knee extension, isokinetic pedaling peak torque (IPT) (30° and 210°/s slow and fast velocities, respectively), ROM of the knee joint, muscle soreness assessed using a visual analog scale (VAS), the circumference of the thigh, and echo intensity, muscle thickness, and shear elastic modulus using the ultrasonic scanner. VAS, echo intensity, muscle thickness, and shear elastic modulus were muscles of target the vastus lateralis, vastus medialis, and rectus femoris. These measurements were performed before, immediately after and 1 and 4 days after the ECCs cycling exercise. All subjects attended a familiarization session at least 1 week before the exercise where the subjects were briefed on eccentric exercise protocols. In the familiarization session, the subjects practiced for 3 min with a very light load of cycling exercise in ECCs mode similar to the present experiment.

### Eccentric cycling

The velocities of the ECCs cycling exercise were either 30°/s (5 rpm; Slow) or 210°/s (35 rpm; Fast) using a cycle ergometer (Strength Ergo 240 BK-ERG-003, Mitsubishi Electric Engineering, Tokyo, Japan). The cycling time of 5 min for the Fast was set based on a previous study (Elmer et al., 2010; Ueda et al., 2020). After the Fast, the Slow reached when the total work done was equal to that generated during the Fast. This ergometer was controlled by a servo motor which could be programmed with various exercise programs using a personal computer. For the testing position, the recumbent position was set at a seat angle, i.e., the angle between the backrest and the seat was set to 105°, and the pedal shaft was set at 55 cm from ground level (Kato et al., 2011). The subjects were secured with seat belts for safety. The left and right cranks and pedals of the ergometer were all set to the fixed mode, which enabled the subjects to put their feet on the cleated shoes fitted on the pedals and then generate exercise of the dorsal or plantar flexion of the right ankle joint. The exercise starting positions of the cranks, pedals, and seat were adjusted for enabling the subjects to maintain a comfortable and fixed posture. The subjects were asked to perform all bouts of exercise using either the right or left lower limb (hip and knee joint at 45° of flexion; ankle joint at 0° of plantar/dorsal flexion) and to relax the other lower limb (hip and knee joint at 0° of flexion/extension; relaxed ankle joint) throughout the experiments (Liang et al., 2011; Ueda et al., 2020). The non-exercising leg was secured to a stabilization platform. The range of motion of the knee joint during cycling ranged from about 20° to 120° (0°, full extension). The mechanical work performed during cycling were recorded at a 10-Hz sampling rate in a computer connected to the cycle ergometer (Strength Ergo 240 BK-ERG-003, Mitsubishi Electric Engineering, Tokyo, Japan).

### Maximal voluntary isometric contraction (MVC) torque of knee extension

For the measurement of MVC torque of knee extension, the participants performed the two times 3-s MVCs at knee joint angles of 90° with a 60-s rest between the contractions. The peak torque was considered as the MVC torque of knee extension. The torque signal was amplified using a strain amplifier (LUR-A-100NSA1; Kyowa Electronic Instruments, Tokyo, Japan). The analog torque signal was converted to digital signal using a 16-bit analog-to-digital converter (Power-Lab 16SP; AD Instruments, Bella Vista, Australia). The sampling frequency was set at 10 kHz. The measurement was performed as previously described (Sasaki et al., 2011).

### Isokinetic pedaling peak torque (IPT)

IPT torque of pedaling was applied by measured in a cycle ergometer which is a device similar to the one used for performing eccentric cycling (Strength Ergo 240 BK-ERG-003, Mitsubishi Electric Engineering, Tokyo, Japan). For the measurement of IPT torque of pedaling, the subject performed two three pedals IPT at 30° and 210°/s with a 60-s resting period between contractions. The peak torque of each velocity was used as the IPT.

### Muscle soreness

Muscle soreness was assessed using a 10-cm VAS in which 0 indicated “no pain” and 10 indicated “the worst pain imaginable”; the subject indicated his pain level on this VAS scale. Muscle soreness was assessed by pressure, using a digital muscle stiffness instrument (NEUTONE TDM-NA1, Satou Shouji Inc., Kanagawa, Japan) on vastus lateralis, rectus femoris, and vastus medialis. The pressure was applied perpendicularly to the point on each muscle. The pressures were applied to the vastus lateralis and rectus femoris at the lateral femoral epicondyle and 50% of the greater trochanter, and the vastus medialis at the lateral femoral epicondyle and 30% distal to the greater trochanter. All tests were conducted by the same investigator who had practiced applying the same pressure over time and on different participants.

### Range of motion

Range of motion was determined as the difference in the joint angles between maximal voluntary flexion and extension of the knee joint using a goniometer (Takase Medical, Tokyo, Japan). The flexion was measured when the subject attempted to maximally flex the knee joint of the exercised leg to touch his hip with his heel while keeping the knee joint aligned to the standing leg and supporting the body by placing both hands on the wall, 30 cm from the foot. The extension was measured when the subject attempted to extend the knee joint of the exercised leg as much as possible. ROM was calculated by subtracting the flexion from extension of the knee joint (Chen et al., 2011; Chen et al., 2013; Ueda et al., 2020).

### Circumference

When each subject stood with his feet approximately 10 cm apart, with his body weight evenly distributed on both feet, the perimeter distance of the thigh perpendicular to the long axis of the femur at the marked mid-trochanterion-tibiale level was measured (Chen et al., 2011). The measurements were performed thrice for each time point, and the average of the three measurements was used for further analysis.

### Muscle stiffness, muscle thickness, and echo intensity

Using ultrasound shear wave elastography, we measured muscle stiffness at vastus lateralis, rectus femoris, and vastus medialis with the probe placed at the position (the vastus lateralis and rectus femoris at the lateral femoral epicondyle and 50% of the greater trochanter, and the vastus medialis at the lateral femoral epicondyle and 30% distal to the greater trochanter) marked for the circumference measurement. An ultrasonic scanner (Aixplorer version 4.2, Supersonic Imagine, France) was used in shear wave elastography mode with a musculoskeletal preset. An electronic linear array probe (SL15-4, Supersonic Imagine France) coated with water soluble transmission gel was placed longitudinally on each muscle head. Muscle shear modulus (*μ*), a measure of normalized muscle stiffness was calculated using the following equation: *μ* = *ρV*s^2^, where *ρ* is the density of muscle (assumed to be 1,000 kg/m^3^) and *V*s. is the velocity of shear wave propagation caused by the focused ultrasound beam from the scanner. A 10-mm square map of the muscle shear modulus with a spatial resolution of 1 × 1 mm^2^ was obtained with each ultrasound image. We calculated the average muscle stiffness by combining the measurements obtained for vastus lateralis, rectus femoris, and vastus medialis (Lacourpaille et al., 2017). A representative value of the shear modulus for each muscle head was then determined via spatial averaging over a 5-mm diameter circle (Ochi et al., 2018). Scanned images of each muscle were transferred to a personal computer and the thicknesses of the vastus lateralis, rectus femoris, and vastus medialis were manually calculated by tracing each muscle using image analysis software (ImageJ, MD, United States). To measure the echo intensity, the gains and contrast were kept consistent over the experimental period. The transverse images were analyzed in a computer, in bitmap (.bmp) format. The average echo intensity for the region of interest (20 × 20 mm) was calculated using ImageJ software that provided a grayscale histogram (0, black; 100, white) for the region, as described in a previous study (Tsuchiya et al., 2019). The echo intensity and muscle thickness were evaluated at the same locations as muscle stiffness.

### Statistical analyses

All analyses were performed using the SPSS software version 27.0 (IBM Corp., Armonk, NY, United States). Values are expressed as means ± SD. Exercise time, energy expenditure, mechanical work, peak torque performed during eccentric cycling, and the baseline data for all outcomes at Fast and Slow were compared using the paired t-test. Time courses of MVC torque of knee extension, IPT of pedaling, ROM, circumference, shear elastic modulus, muscle thickness, and echo intensity of values were calculated based on relative changes from the baseline. MVC torque of knee extension, IPT of pedaling, ROM, muscle soreness, echo intensity, muscle thickness, and shear elastic modulus were compared between the Fast and Slow groups via two-way repeated-measure analysis of variance (ANOVA). When a significant main effect or interaction was detected, Bonferroni’s correction was performed for the *post hoc* testing. A *p* < 0.05 was considered statistically significant.

## Results

### During ECCs cycling

As shown in Table 1, a significant difference in exercise time was observed in the Slow longer than Fast. However, a significant difference was not observed in the mechanical work between Slow and Fast.

**TABLE 1:** Means values (SD) during eccentric cycling for the Fast and Slow, exercise time, energy expenditure, total mechanical work, and peak torque at 210°/s, and 30°/s. *Denotes a significant (*p* < 0.05) difference between Fast and Slow.

	Fast (210 deg/sec)	Slow (30 deg/sec)
Exercise times (sec)	300.0 ± 0.0	1422.0 ± 330.0 *
Mechanical work (J)	14897.1 ± 2747.1	14868.9 ± 2763.0
Mechanical work/body mass (J/kg)	214.8 ± 42.4	214.3 ± 42.2

*Denotes a significant (*p* < 0.05) comparison whit Fast.

### Maximal voluntary isometric contraction (MVC) torque of knee extension

**FIGURE 1:**
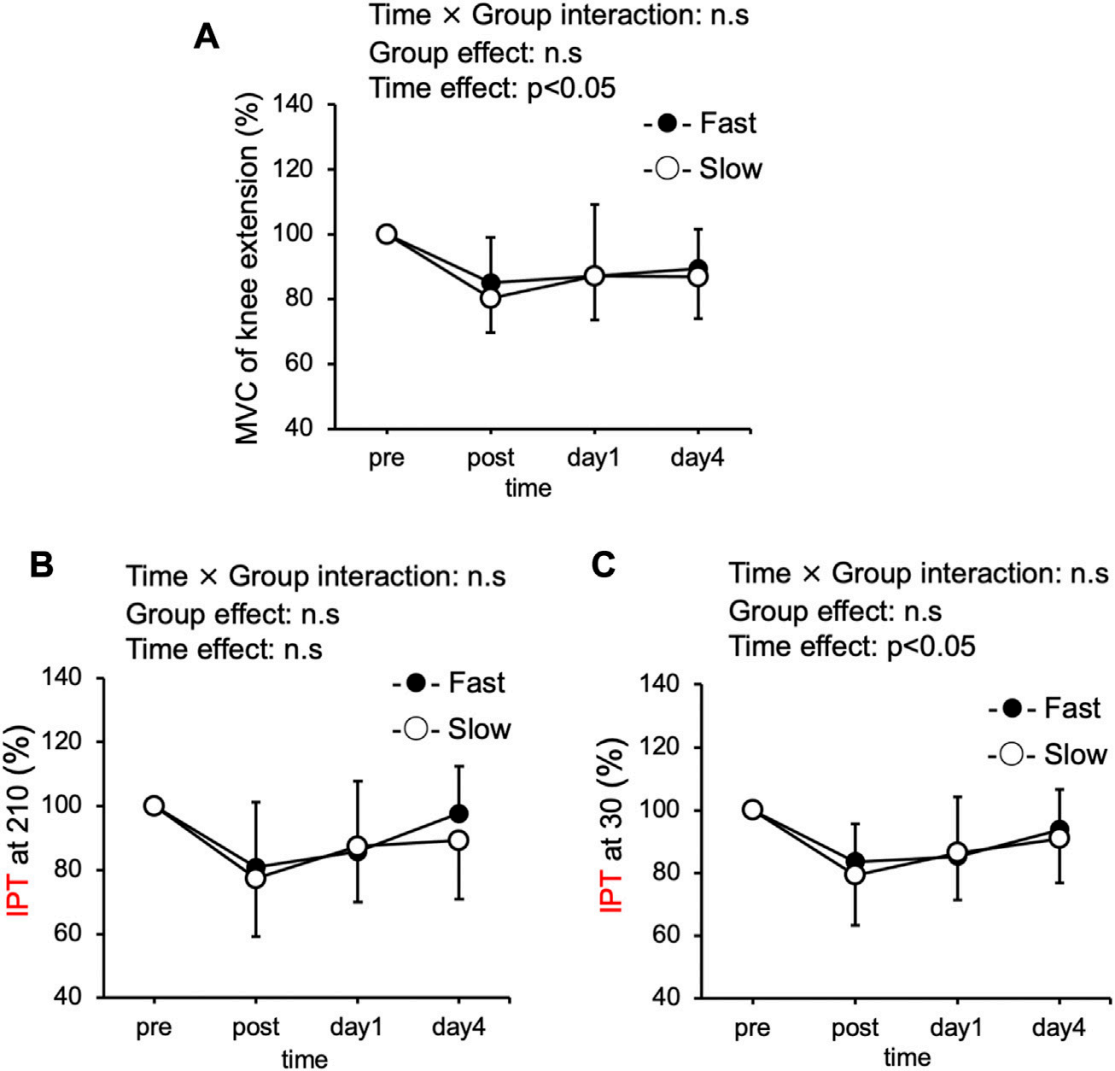
Changes (mean ± SD) in maximal voluntary isometric contraction (MVC) torque of knee extension **(A)**, and isokinetic pedaling peak torque (IPT) torque of pedaling at 210°/s **(B)**, and 30°/s **(C)**, before (pre), immediately after (post), 1 day, and 4 days after exercise in the slow velocity session (Slow) and fast velocity session (Fast).

Significant interaction effect was not observed in the MVC torque of knee extension between the Fast and Slow groups are shown in Figure 1A. However, a significant time effect was found at the MVC torque of knee extension. MVC torque of knee extension at the baseline was similar between the two groups (Fast: 1.7 ± 0.4 Nm/kg; Slow: 1.8 ± 0.5 Nm/kg). Compared with the pre-exercise value, MVC torque in both groups significantly decreased immediately after exercise and remained decreased up to 4 days after exercise (*p* < 0.05).

### Isokinetic pedaling peak torque (IPT)

Significant interaction effect was not observed in IPT torque of pedaling at 210°/s between Fast and Slow groups. IPT of pedaling at 210°/s at the baseline was the same between the two groups (Fast: 1.9 ± 0.35 Nm/kg; Slow: 2.0 ± 0.42 Nm/kg) (Figure 1B). The significant interaction effect was not observed in IPT of pedaling at 30°/s between Fast and Slow groups. However, a significant time effect was found at IPT of pedaling at 30°/s. IPT of pedaling at 30°/s at the baseline was similar between the two groups (Fast: 2.4 ± 0.5 Nm/kg; Slow: 2.5 ± 0.4 Nm/kg). Compared with the pre-exercise value, IPT at 30°/s in the Fast significantly decreased immediately after exercise and remained decreased up to 1 day after exercise, but the Slow significantly decreased immediately after exercise and remained decreased up to 4 days after exercise (*p* < 0.05) (Figure 1C).

### Muscle soreness

**FIGURE 2:**
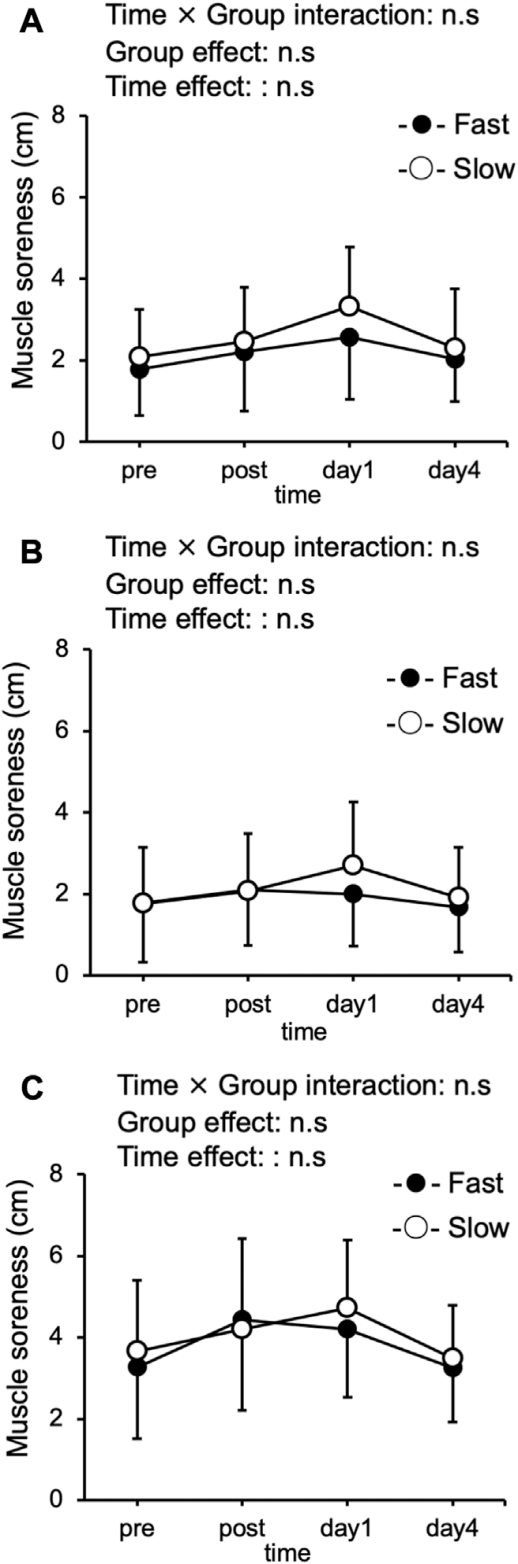
Changes (mean ± SD) in muscle soreness were recorded using a visual analog scale for the vastus lateralis **(A)**, rectus femoris **(B)**, and vastus medialis **(C)** immediately after (post), 1 day, and 4 days after exercise in the slow velocity session (Slow) and fast velocity session (Fast).

Significant interaction effect was not observed in muscle soreness (Figure 2). Muscle soreness in three muscle groups at the baseline was similar in the two conditions (vastus lateralis: Fast: 1.8 ± 1.1 cm, Slow: 2.1 ± 1.2 cm; rectus femoris: Fast: 1.8 ± 1.5 cm, Slow: 1.8 ± 1.4 cm; vastus medialis: Fast: 3.3 ± 1.7 cm, Slow: 3.7 ± 1.9 cm). Compared with the pre-exercise values was not significant difference after exercise at any time points in vastus lateralis, rectus femoris, and vastus medialis in both conditions.

### Range of motion, circumference, and muscle thickness

**FIGURE 3:**
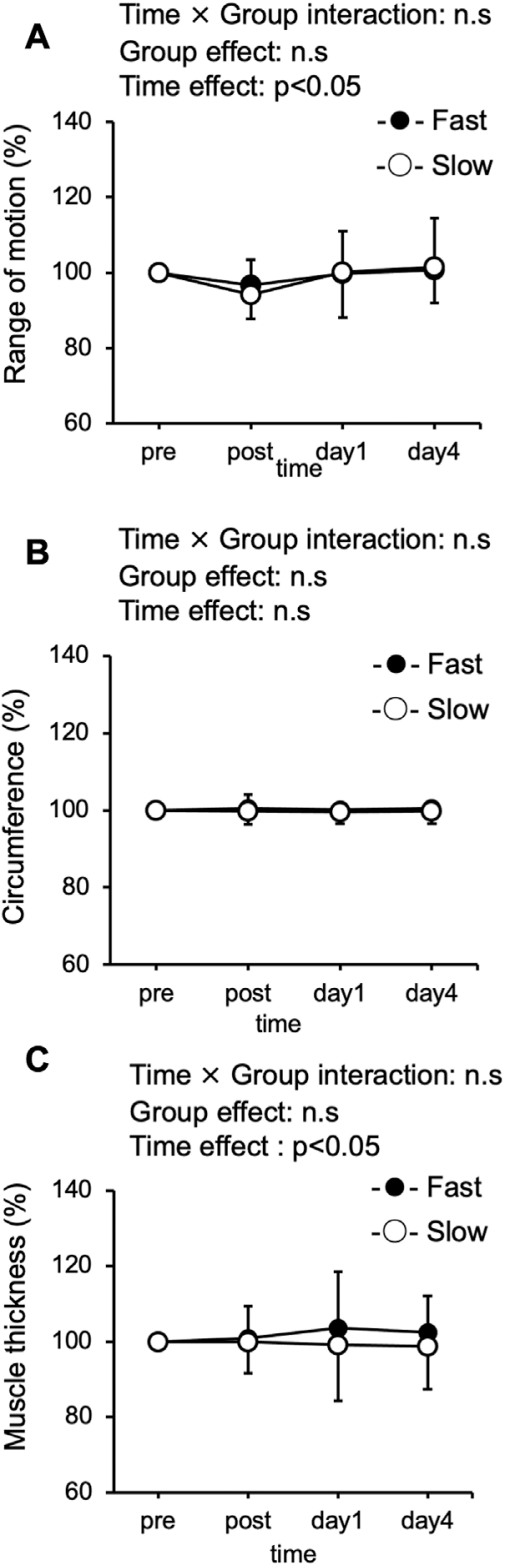
Changes (mean ± SD) in range of motion **(A)**, thigh circumference **(B)**, and muscle thickness **(C)**, immediately after (post), 1 day, and 4 days after exercise in the fast velocity session (Fast) and slow velocity session (Slow).

Figure 3A showed that significant interaction effect was not observed in the range of motion between the Fast and Slow conditions. However, a significant time effect was found in ROM. ROM at the baseline was similar between the two groups (Fast: 112.4° ± 9.7°, Slow: 110.5° ± 8.93°). Compared with the pre-exercise value, ROM in the Slow significantly decreased only immediately after exercise (*p* < 0.05). On the other hand, ROM in the Fast was not significant after exercise at any time point compared to before exercise value. A significant interaction effect was not observed in circumference between the Fast and Slow conditions (Figure 3B). The circumference at the baseline was similar between the two groups (Fast: 54.0 ± 6.5 cm, Slow: 54.6 ± 6.8 cm). Compared with the pre-exercise value, circumference in both conditions was not significant difference after exercise at any time point. A significant interaction effect was not observed in muscle thickness between the Fast and Slow conditions. However, a significant time effect was found in muscle thickness (Figure 3C). The muscle thickness at the baseline was similar between the two groups (Fast: 9.2 ± 1.4 cm, Slow: 9.4 ± 1.5 cm). Compared with the pre-exercise value, muscle thickness in both conditions was not significant difference after exercise at any time point.

### Echo intensity and muscle stiffness

**FIGURE 4:**
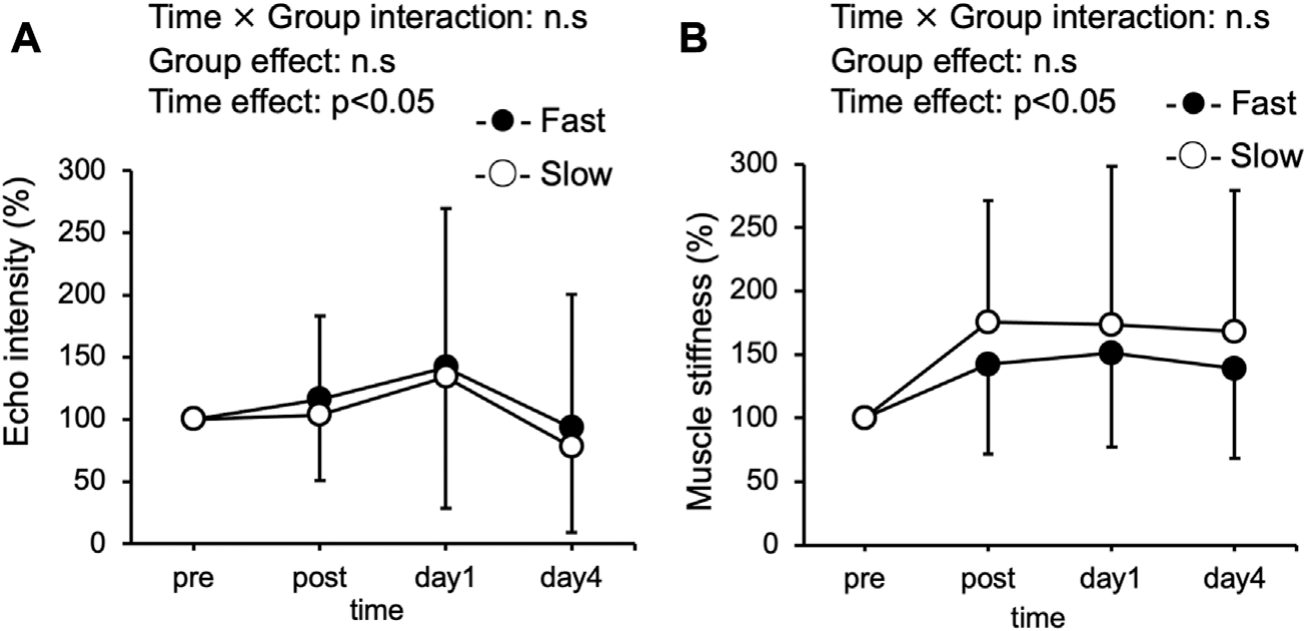
Changes (mean ± SD) in echo intensity of the quadriceps **(A)**, and muscle stiffness of the knee extensor (vastus laterials, rectus femoris, and vastus medialis) **(B)**, before (pre), immediately after (post), 1 day, and 4 days after eccentric muscle actions in the slow velocity session (Slow) and fast velocity session (Fast).

A significant interaction effect was not observed in echo intensity between the Fast and Slow conditions (Figure 4A). Although there was a significant time effect for echo intensity, both conditions were no significant difference at any time point after exercise compared to pre-exercise values. The echo intensity at the baseline was similar between the two groups (Fast: 32.9 ± 17.0, Slow: 34.1 ± 17.3). A significant interaction effect was not observed in muscle stiffness between the Fast and Slow conditions (Figure 4B). However, a significant time effect was found in muscle stiffness. The muscle stiffness at the baseline was the same between the two conditions (Fast: 14.2 ± 6.8 kPa, Slow: 15.6 ± 8.7 kPa). Compared with the pre-exercise value, muscle stiffness in the Slow significantly increased immediately after exercise and remained increased up to 4 days after exercise (*p* < 0.05). On the other hand, muscle stiffness in the Fast was not significant after exercise at any time point compared to before exercise value.

## Discussion

The present study compared the muscle damage caused by fast (210°/sec) and slow (30°/sec) ECCs cycling, under equal mechanical work conditions. The results showed absence of difference in muscle damage owing to the difference in pedaling velocity, although torque deficit and delayed onset muscle soreness were observed in both conditions. These results supported our hypothesis.

In the present study, the slow condition was performed until the mechanical work was equal to that obtained in the fast condition, so the slow (1422.0 ± 330.0 s) movement time was much longer than the fast (300.0 ± 0.0 s) (Table 1). On the other hand, the present study monitored the accumulated mechanical work from the torque output, which made it possible to unify the mechanical work of Fast and Slow during the exercise. In a study comparing the degree of muscle damage caused by differences in contraction speed, the contraction time (120 s) of the ECCs exercise with elbow flexion was unified (Chapman et al., 2006), and in another study, the number of contractions was unified to 30 or 210 times (Chapman et al., 2008a). Both studies showed that the muscle damage was greater in the fast-speed condition than in the slow-speed. However, although these previous studies (Chapman et al., 2006; Chapman et al., 2008a) have standardized the contraction time and number of contractions, they have not standardized the mechanical work. Therefore, the present study is the first to examine the effects of different pedaling velocities on muscle damage under equivalent mechanical work conditions using ECCs cycling.

In the present study, there is no significant interaction between the MVC torque of knee extension and IPT of pedaling at 30 and 210 (Figure 1). A previous study comparing the contraction velocity of the ECCs movement with elbow flexors in the fast speed (210°/s) and low speed (30°/s) conditions reported significantly greater torque deficit in the fast (Chapman et al., 2008a) 2). In our previous study, the exercise duration was standardized to 5 min in ECCs cycling, and the results were compared between the fast (210°/s) and slow (30°/s) conditions. The results of this study showed that the torque deficit was significantly greater in the fast (Ueda et al., 2020). Furthermore, in a previous study in which mechanical work was calculated by the number of repetitions and contraction time of ECCs in elbow flexion, it was reported that the mechanical work had no effect on muscle strength loss during ECCs (Chapman et al., 2008b). From these previous studies, it is concluded that contraction velocity during exercise is strongly related to muscle strength loss after ECCs. Contrarily, Mavropalias et al. (2020) compared the muscle damage before and after ECCs cycling at high (20% of peak power for 1 min x five sets) and low intensities (5% of peak power for 4 min x five sets). They reported that a significant difference in IPT of pedaling at 90°/s in both conditions was absent, despite the 4-fold difference in exercise intensity. Although differences in contraction velocity were not examined, it is suggested that mechanical work may be related to post-exercise muscle weakness in ECCs cycling. In light of these previous studies and the results of the present study, we suggest that the degree of muscle strength loss may be dependent on mechanical work than on velocity during ECCs cycling under equal mechanical work conditions.

The delayed onset of muscle soreness in the present study did not significantly differ between the conditions (Figure 2). In our previous study (Ueda et al., 2020) showed that the delayed onset of muscle soreness of the rectus femoris muscle and medial vastus medialis was significantly higher in the fast (210°/sec) than in slow (30°/sec). However, in this study, the mechanical work during exercise was significantly greater in the fast than in the slow. Regarding the relationship between mechanical work and delayed onset muscle soreness, Paschalis et al. (2005) performed ECCs in knee extension in the high intensity condition (10 × 12 sets at maximal effort) followed by the low intensity condition. The results showed that muscle soreness in both conditions was comparable to that in the high-intensity condition. The results reported absence of difference in the muscle soreness between the two conditions, suggesting that mechanical work may be a more important factor for muscle soreness than exercise intensity (Paschalis et al., 2005). Although this study did not examine differences in velocity, the involvement of mechanical work in delayed onset of muscle soreness after ECCs supports the results of the present study. These results suggest that delayed onset muscle soreness after ECCs cycling may be similar when mechanical work is unified even at different contraction velocities and exercise intensities.

It has been reported that the fast velocity (210°/s) ECCs of elbow flexion, i was associated with significantly greater ROM limitation and muscle swelling than the slow velocity (30°/s) (Chapman et al., 2006; Chapman et al., 2008a). Contrarily, in our previous study (Ueda et al., 2020) and present study, differences in ROM and muscle swelling between the fast (210°/s) and slow (30°/s) speed conditions were not observed after the 5 min of ECCs cycling. In the present study, muscle stiffness and echo intensity were also assessed in order to evaluate the condition of skeletal muscle. Changes in muscle stiffness after ECCs have been reported to reflect a rapid disruption of calcium homeostasis after exercise-induced myofibrillar destruction (Lacourpaille et al., 2014; Lacourpaille et al., 2017). It has also been speculated that the increase in echo intensity reflects the influx of water into the muscle (Nosaka and Clarkson, 1996). Similar to the present study, significant differences in changes in muscle stiffness and echo intensity during 5 min of ECCs cycling in the fast (210°/s) and slow (30°/s) conditions were absent in our previous study (Ueda et al., 2020). Future studies should clarify the mechanism in more detail.

The present study has several limitations that should be considered. First, although the mechanical work is matched in this study, it is unclear whether the average power output in ECCs cycling was consistent in both conditions. Further study is necessary to examine the effects of not only the mechanical work and pedaling speed but also the factor of power output on muscle damage. Second, the mechanical work of the Fast condition was smaller than the mechanical work of our previous study under similar exercise conditions (Ueda et al., 2020). The baseline IPT of pedaling at 30°/s was smaller in the present study (169.8 ± 42.9 Nm) than in the previous study (205.4 ± 35.9 Nm). Therefore, it is likely that the present study had different muscular characteristics of the subjects compared to the previous study (Ueda et al., 2020). Third, only two exercise conditions were used in the present study. Future studies should examine the effects of mechanical work and contraction velocity on muscle damage in detail under multiple velocity conditions and exercise loads that cause more severe damage.

## Conclusion

The results of this study suggest that muscle damage by ECCs cycling may be affected by mechanical work more than contraction velocity. The present study may provide useful information for determining the speed conditions for ECCs cycling training. A recent review article (Barreto et al., 2021) has stated that the development of muscle damage should be considered in ECCs cycling training program. In particular, it is paramount to avoid DOMS during the training program in rehabilitation settings. We believe that our results suggest that low pedal cadence may be safer in rehabilitation settings, while high pedal cadence may be more efficient when applying large loads in a short time. Future studies should examine mechanical work, pedaling velocity, and long-term training adaptation.

## Data Availability

The raw data supporting the conclusions of this article will be made available by the authors, without undue reservation.
